# Supplementary material for: Lid loop-mediated proton transfer revealed in the Fe/αKG-dependent decarboxylase TraH
Source: Commun Chem. 2026 Apr 14;9:159. doi: 10.1038/s42004-026-01986-9 (PMC13083874; doi:10.1038/s42004-026-01986-9)
Supplement: Supplementary file 2 — support information [file 42004_2026_1986_MOESM2_ESM.pdf]

Support information

Lid loop-mediated proton transfer revealed in the Fe/αKG-dependent decarboxylase TraH

Xuehua Zheng[a,b,#], Rui Ge[c,#], Zhiyong Guo[d,#], Mathias Girbig[e,f], Johannes Freitag[a], Aitao Li[d], Georg K. A. Hochberg[e,f], Shu-Ming Li[c,*], Gert Bange[a,e,*], Liujuan Zheng[a,e,*]

[a]*Philipps-Universität Marburg, Center for Synthetic Microbiology (SYNMIKRO) & Department of Chemistry, Karl-von-Frisch Strasse 14, 35043 Marburg, Germany*

[b]*Guangzhou Municipal and Guangdong Provincial Key Laboratory of Molecular Target & Clinical Pharmacology, the NMPA and State Key Laboratory of Respiratory Disease, School of Pharmaceutical Sciences, Guangzhou Medical University, Guangzhou 511436, China*

[c]*Philipps-Universität Marburg, Fachbereich Pharmazie, Institut für Pharmazeutische Biologie und Biotechnologie, Robert-Koch Straße 4, 35037 Marburg, Germany*

[d]*State Key Laboratory of Biocatalysis and Enzyme Engineering, Hubei Key Laboratory of Industrial Biotechnology, School of Life Sciences, Hubei University, Wuhan 430062, China*

[e]*Max-Planck Institute for Terrestrial Microbiology, Karl-von-Frisch Strasse 14, 35043 Marburg, Germany*

[f]*Philipps-Universität Marburg, Department of Biology, Karl-von-Frisch Strasse 1, 35043 Marburg, Germany*

[#] These authors contribute equally to this work.

[*] Corresponding authors: shuming.li@staff.uni-marburg.de, gert.bange@synmikro.uni-marburg.de, liujuan.Zheng@mpi-marburg.mpg.de

Table S1. Data Collection and Refinement Statistics

	TraH•Mn^{2+}•αKG•1b	TraH•Mn^{2+}•αKG•3b	TraH•Mn^{2+}
	**9IG5**	**9IG3**	**9IG4**
**Data collection**			
Wavelength (Å)	0.97626	0.97626	0.97626
Resolution (Å)	49.15 - 2.4 (2.55 - 2.40)	33.97 - 2.89 (3.07 - 2.89)	49.06 - 2.1 (2.23 - 2.10)
Space group	$P4_32_12$	$P4_32_12$	$P4_32_12$
a, *b*, *c* (Å)	77.21, 77.21, 225.87	76.35, 76.35, 223.35	77.20, 77.20,223.40
α, β, γ (°)	90, 90, 90	90, 90, 90	90, 90, 90
Total reflections	677695 (118233)	377395 (57876)	1053145 (177370)
Unique reflections	50560 (8201)	28044 (4514)	75250 (12176)
Multiplicity	13.4 (14.3)	13.5 (12.8)	14.0 (14.6)
Completeness (%)	99.2 (99.3)	99.8 (99.6)	100 (99.9)
Mean I/sigma(I)	13.45 (2.38)	7.19 (2.35)	12.23 (2.40)
R_{merge}	0.1707 (1.109)	0.3792 (1.123)	0.1268 (1.121)
$CC_{1/2}$	0.998 (0.774)	0.992 (0.650)	0.999 (0.841)
**Refinement**			
R_{work}/R_{free}	0.1928/ 0.2545	0.2399/ 0.2927	0.1973 / 0.2353
No. of atoms			
macromolecules	4780	4689	4495
ligands	85	49	2
solvent	137	0	205
R.m.s. deviations			
Bond lengths (Å)	0.008	0.003	0.007
Bond angles (°)	0.95	0.53	0.89
Ramachandran favored (%)	95.63	88.26	95.99
B factors ($Å^2$)			
macromolecules	39.78	41.20	45.95
Gln97-Asn102/A	43.57	53.37 (97-99)	69.55 (97-98)
Asp112-Lys114/A	44.85	54.88 (113-114)	57.75 (113-114)
ligands			
Mn^{2+}/A	35.16	53.09	40.82
αKG/A	34.85	70.135	-
substrate/A	32.94	39.10	-
solvent	37.94	-	43.51

Table S2. Mutations Designed to Probe the Hydrogen-bond Network in TraH

Residue	Mutant	Design Considerations
**Q99**	Q99E	Introduces a negative charge and altered side-chain geometry, disrupting the native hydrogen-bonding interactions and potentially introducing electrostatic repulsion.
	Q99S	Retains hydrogen-bonding capability but lacks the amide functionality and side-chain volume of glutamine, probing the importance of specific geometry and steric bulk.
	Q99K	Introduces a positively charged side chain with distinct geometry, testing the effects of charge inversion and altered side-chain conformation.
	Q99L	Removes hydrogen-bonding capability while largely preserving side-chain volume, allowing steric effects to be evaluated independently of hydrogen-bonding interactions.
	Q99R	Introduces a long, flexible guanidinium group to assess whether an alternative positively charged, hydrogen-bonding side chain can functionally substitute for glutamine.
	Q99H	A conservative substitution that partially preserves hydrogen-bonding capability and side-chain volume, representing the closest chemical analogue to glutamine among the tested variants.
**D112**	D112L	Eliminates the negative charge and hydrogen-bonding capability of the carboxylate while approximately maintaining steric bulk.
**K114**	K114R	Preserves the positive charge while altering side-chain length and hydrogen-bonding geometry, probing the specific requirement for the lysine ε-amino group.
	K114M	Removes both the positive charge and hydrogen-bonding potential, testing the necessity of electrostatic interactions at this position.
**K191**	K191R	Maintains a positive charge but replaces the ammonium group with a guanidinium moiety, altering hydrogen-bonding patterns to probe the precision required for electrostatic and geometric complementarity.
**Y214**	Y214F	Removes the phenolic hydroxyl group while retaining the aromatic ring, isolating the contribution of the hydroxyl-mediated hydrogen-bonding interactions.
	Y214H	Replaces the phenolic hydroxyl with an imidazole group, altering hydrogen-bonding character and pKa to probe sensitivity to local chemical environment.
**Negative controls**	E98A, Q100A, S208A	Proximal loop residues not directly involved in the hydrogen-bond network; included as controls to distinguish specific network disruption from global structural perturbation.

Table S3. Average Active Site Distances (Å) in Key States of the TraH-Catalyzed Decarboxylation of Crustosic Acid

	RC	TS1	INT1	TS2	INT2	TS3	P
Fe–O1	1.64 ± 0.03	1.82 ± 0.11	1.89 ± 0.10	1.90 ± 0.06	2.01 ± 0.08	2.22 ± 0.22	2.46 ± 0.34
H5–C5	1.09 ± 0.04	1.33 ± 0.08	2.75 ± 0.10	-	-	-	-
H5–O1	2.25 ± 0.06	1.34 ± 0.08	0.98 ± 0.09	-	-	-	-
C6–C5	1.54 ± 0.02	1.53 ± 0.05	1.45 ± 0.04	1.39 ± 0.02	1.34 ± 0.03	-	-
C6–C7	-	-	1.64 ± 0.07	2.26 ± 0.05	3.44 ± 0.03	-	-
H1–O_W	-	-	-	-	1.00 ± 0.04	1.13 ± 0.05	1.95 ± 0.06
H1–O1	-	-	-	-	1.96 ± 0.05	1.41 ± 0.05	0.97 ± 0.15

The structures of all states were derived from the calculated 1D-profile of the decarboxylation reaction pathway. Refer to Figure **5**, **S5**, and **S6** for visualization.

Table S4. Representative Spin Densities (a.u.) along the TraH-Catalyzed Decarboxylation of Crustosic Acid

Species	Fe	O1	C5	others
**MC**	3.27	0.58	0.00	0.16
**TS1**	3.34	0.24	0.02	0.41
**INT1**	3.43	0.10	0.48	-0.01
**TS2**	3.51	0.03	0.00	0.45
**INT2**	3.92	0.09	0.00	-0.01
**P**	3.77	0.00	0.00	0.23

Table S5. Dali Analysis of Structural Similarity between TraH and Related Enzymes

No.	PDB ID	Z-score	RMSD (Å)	Identity (%)	Aligned Residues	Description
1	6XJJ	34.9	1.9	41	281	TropC
2	5C3R	33.1	2.5	26	288	T7H
3	6JYV	32.7	2.4	32	284	Unreported
4	5V2Z	31.0	2.5	25	287	EFE
5	1BK0	29.9	2.8	23	287	IPNS
6	3OOX	29.3	2.7	28	280	Unreported
7	6LSV	29.2	2.6	25	277	JOX
8	7EKD	29.0	2.8	22	283	G3OX2
9	6KU3	28.9	2.6	25	271	G2OX3
10	5O7Y	27.9	2.8	22	282	T6ODM
11	8CVC	27.8	3.0	24	276	H6H
12	6KUN	27.8	2.7	30	264	DAO
13	8Y4U	27.7	2.8	23	273	HIS1
14	1GP6	27.3	3.2	26	284	ANS
15	4XAE	26.6	2.9	29	272	H6H
16	6KWA	26.0	2.9	24	253	DAO
17	7E38	25.6	3.1	23	266	DPS
18	1UNB	23.8	2.9	19	246	DAOCS

Table S6. Lid Loop Characteristics in IPNS Subfamily with Available Crystal Structures

No	PDB ID	Uniprot Accession	Name	Function	Length	Conformation	Origin
1	7E38	A0A0N9HQ36	DPS	Cyclization	5	Open	Plant
2	6KUN	Q01IX6	DAO	Oxidation	7	Open	Plant
3	6KWA	Q9XI75	DAO	Oxidation	7	Open	Plant
4	1BK0	P05326	IPNS	Cyclization	10	Open	Fungi
5	6KU3	Q8S0S6	G2OX3	Hydroxylation	11	Slightly open	Plant
6	1GP6	Q96323	ANS	Hydroxylation	14	Slightly open	Plant
7	5V2Z	P32021	EFE	Decarboxylation	14	Closed	Bacteria
8	4XAE	Q9LHN8	H6H	Hydroxylation	15	Slightly open	Plant
9	6JYV	Q9HWJ0	-	Unreported	15	Open	Bacteria
10	6LSV	Q9FFF6	JOX	Hydroxylation	15	Slightly open	Plant
11	6TTM	Q6EZB3	H6H	Hydroxylation	15	Slightly open	Plant
12	7EKD	Q9FU53	G3OX2	Hydroxylation	15	Slightly open	plant
13	8CVC	Q9XJ43	H6H	Hydroxylation	15	Slightly open	Plant
14	3OOX	Q9ABM7	-	Unreported	16	Open	Bacteria
15	5O7Y	D4N500	T6ODM	Oxidation	16	Open	Plant
16	6XJJ	B8M9K5	TropC	Ring expansion	16	Slightly open	Fungi
17	3ON7	Q8EE01	-	Unreported	17	Open	Bacteria
18	7W5S	A0A1U8X168	AdaV	Halogenation	17	Disorder	Bacteria
19	1UNB	P18548	DAOCS	Ring expansion	20	Disorder	Bacteria
20	5C3R	Q7RYZ9	T7H	Hydroxylation	27	Open	Fungi
21	1W9Y	Q08506	ACCO	Decarboxylase	-	None	Plant
22	8Y4U	Q6ES21	HIS1	Hydroxylation	-	None	Plant
23	5GJ9	Q41931	ACCO	Decarboxylation	-	None	Plant

Table S7. Plasmids and Primers Used in This Study

Plasmids	Description	Primers[a]
pLZ62	pET-24d-Nhis-TraH, a 984 bp fragment of *TraH* from cDNA of *P. crustosum*, with BsaI inserted in pET-24d	TTAAGGTCTCCCATGGGCTCTGTCGATGCGGCCGTCGTCAA
		TTAAGGTCTCCTCGAGCAATGAAGTATCATCCGTCAATCCA
pLZ218	Mutation of Q99H in pLZ62	CCGGGCGAGCATCAAGCCAAC
		GTTGGCTTGATGCTCGCCCGG
pLZ216	Mutation of Q99R in pLZ62	CCGGGCGAGCGACAAGCCAAC
		GTTGGCTTGTCGCTCGCCCGG
pLZ217	Mutation of Q99K in pLZ62	CCGGGCGAGAAACAAGCCAAC
		GTTGGCTTGTTTCTCGCCCGG
pLZ225	Mutation of Q99S in pLZ62	CCGGGCGAGTCACAAGCCAAC
		GTTGGCTTGTGACTCGCCCGG
pLZ193	Mutation of Q99E in pLZ62	CCGGGCGAGGAACAAGCCAAC
		GTTGGCTTGTTCTTCCTCGCCCGG
pLZ227	Mutation of D112L in pLZ62	TTTGAGCGCTTAGCTAAAGAG
		CTCTTTAGCTAAGCGCTCAAA
pLZ194	Mutation of K114M in pLZ62	CGATGCTATGGAGGGTTTCTTTGTGGGAAAGG
		TCATAGCATCGCGCTCAAAGTCACCATG
pLZ232	Mutation of K114R in pLZ62	CGATGCTCGAGAGGGTTTCTTTGTGGGAAAGG
		TCTCGAGCATCGCGCTCAAAGTCACCATG
pLZ195	Mutation of K191M in pLZ62	TTTCTGATGCTACTTCGCTACCCGGCACACA
		TAGCATCAGAAACATTGCAGCGTCTGTGGTAA
pLZ235	Mutation of K191R in pLZ62	TTTCTGCGCCTACTTCGCTACCCGGCACACA
		TAGGCGCAGAAACATTGCAGCGTCTGTGGTAA
pLZ219	Mutation of Y214F in pLZ62	CACACGGATTTCGGCGGCATT
		AATGCCGCCGAAATCCGTGTG
pLZ196	Mutation of Y214H in pLZ62	CACACGGATCATGGCGGCATT
		AATGCCGCCATGATCCGTGTG
pLZ240	Mutation of E98Q in pLZ62	CCGGGCCAACAGCAAGCCAACGACCCACACCA
		TGCTGTTGGCCCGGTCCCTCATAGCCTCGTCG
pLZ241	Mutation of Q100E in pLZ62	GAGCAGGAAGCCAACGACCCACACCATGGTGA
		GTTGGCTTCCCTGCTCGCCCGGTCCCTCATAGC
pLZ228	Mutation of S208A in pLZ62	AAGTTCGGTGCAGGCCAACAC
		GTGTTGGCCTGCACCGAACTT

[a]Primer sequences are provided in the 5'→3' direction, with forward primers (F) in the upper row and reverse primers (R) in the lower row for each pair.

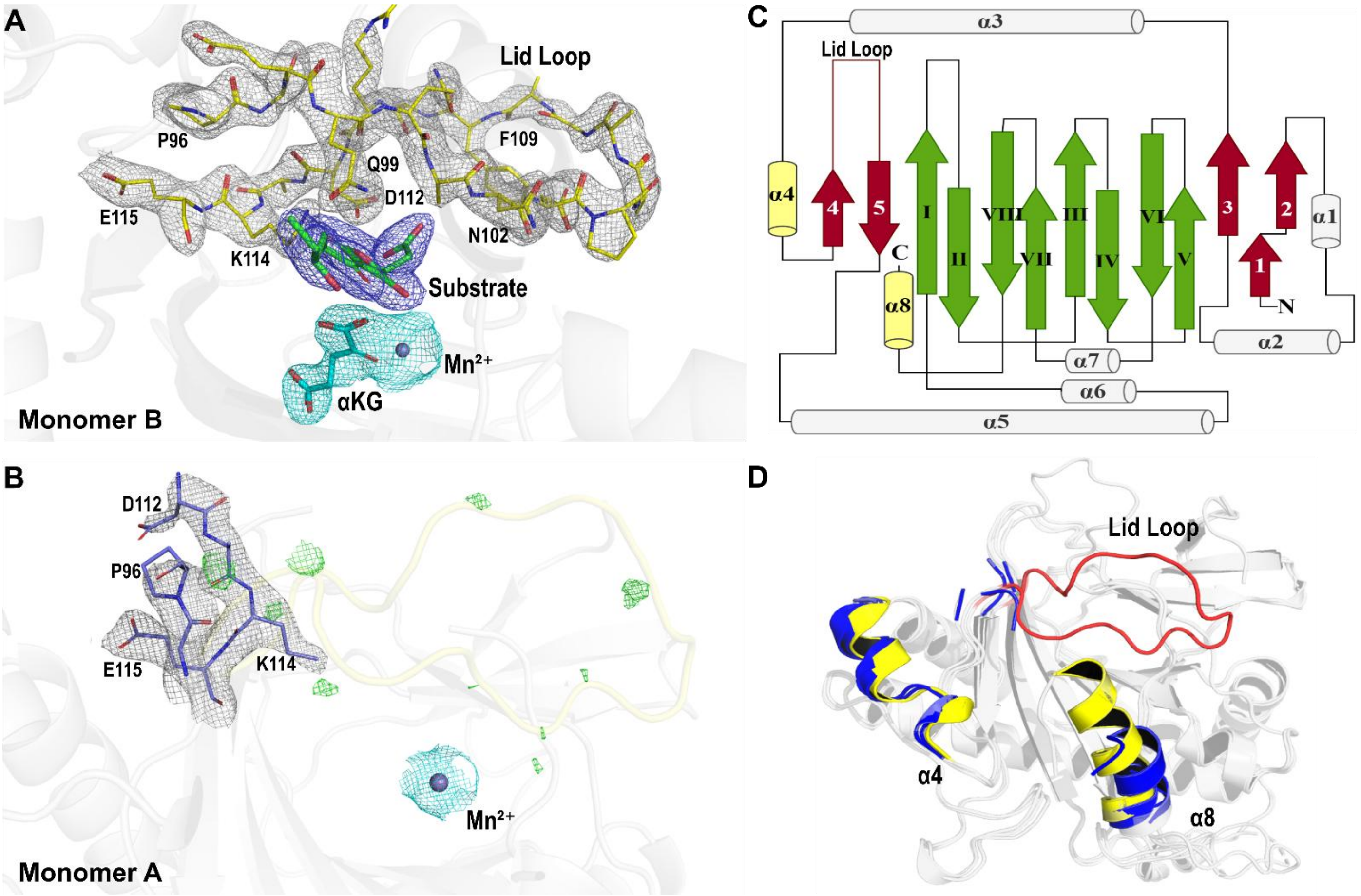

Figure S1. Structural Features and Comparison of TraH Crystal Structure

(A) Electron density map for ligands and lid loop in monomer B. The *2mFo-DFc* map at 1.2 σ (carve = 1.5) for the substrate, co-substrate (Mn^{2+} and αKG), and lid loop is shown in blue, cyan, and gray, respectively.

(B) Electron density map for ligand and lid loop in monomer A. The *2mFo-DFc* map at 1.2 σ (carve = 1.5) for Mn^{2+} and the lid loop is shown in cyan and gray, respectively, while the residual *mFo-DFc* map at 2.5 σ (carve = 1.5) in the lid loop area is shown in green. The lid loop from monomer B is shown in yellow for comparison and to depict the residual map.

(C) Schematic representation of TraH crystal structure. The β-sheets forming the DSBH core are highlighted in green. The N-terminal insertion β-sheets and lid loop are shown in red, while the gate-like helix α4 (from the N-terminal insertion) and α8 (from the C-terminal insertion) are colored yellow.

(D) Comparison of the ligand-bound monomer B with a higher-resolution (2.1 Å) crystal structure of TraH. The gate-like helices α4 and α8 are highlighted in yellow in monomer B, while the lid loop is colored tv-red. In the higher-resolution structure, helices α4, α8, and the lid loop are highlighted in blue.

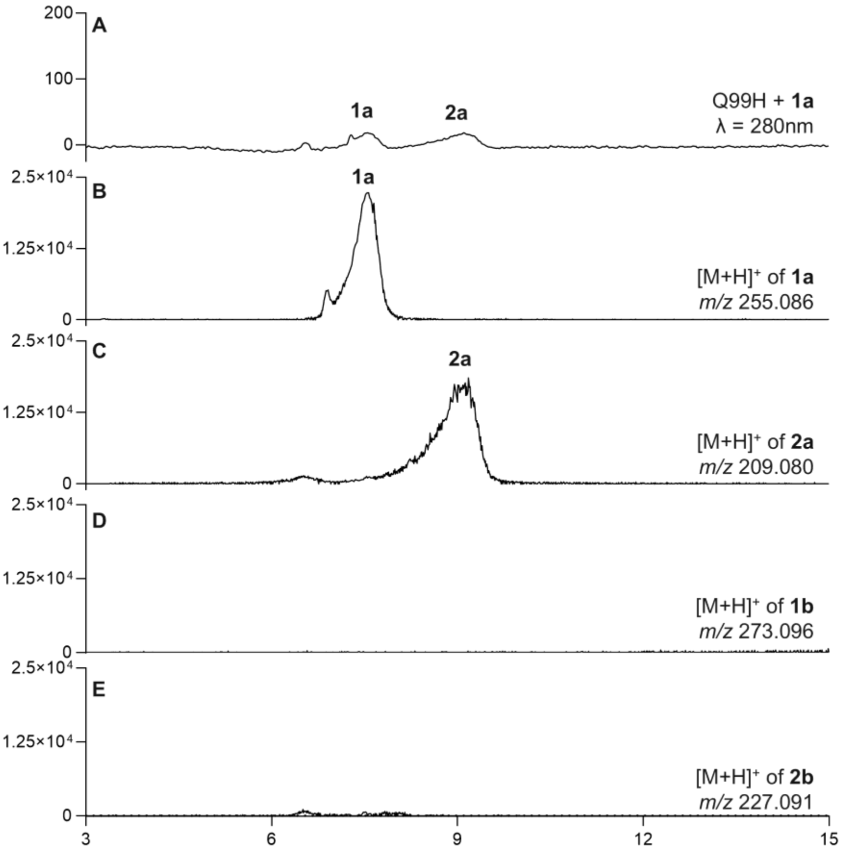

Figure S2. LC-MS analysis of enzyme assays of Q99H with 1a. UV absorption at 280 nm is illustrated **(A)**. EICs refer to $[M + H]^+$ of **1a** at *m/z* 255.086 **(B)**, $[M + H]^+$ of **2** (closed form) at *m/z* 209.080 **(C)**, $[M + H]^+$ of **1b** at *m/z* 273.096 **(D)** and $[M + H]^+$of **2** (open form) at *m/z* 227.091 **(E)** with a tolerance range of ± 0.005.

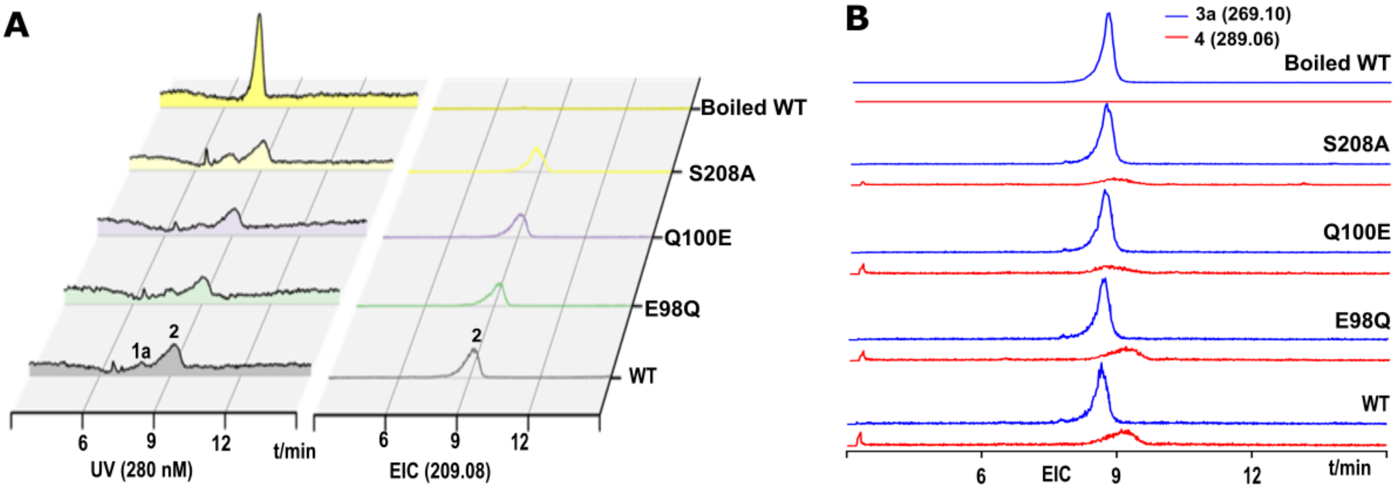

Figure S3. Random Mutations in Lid Loop Residues and Their Effect on TraH-Catalyzed Reactions

Decarboxylation of **1a** or desaturation of **3a** catalyzed by TraH mutants are shown in panel A and panel B, respectively. EICs were extracted for ions at *m/z* 209.08 ($[M+H]^+$), 269.10 ($[M+H]^+$), and 289.06 ($[M+Na]^+$), corresponding to compounds **2**, **3a**, and **4**, respectively. Source data are provided in Supplementary Data 4.

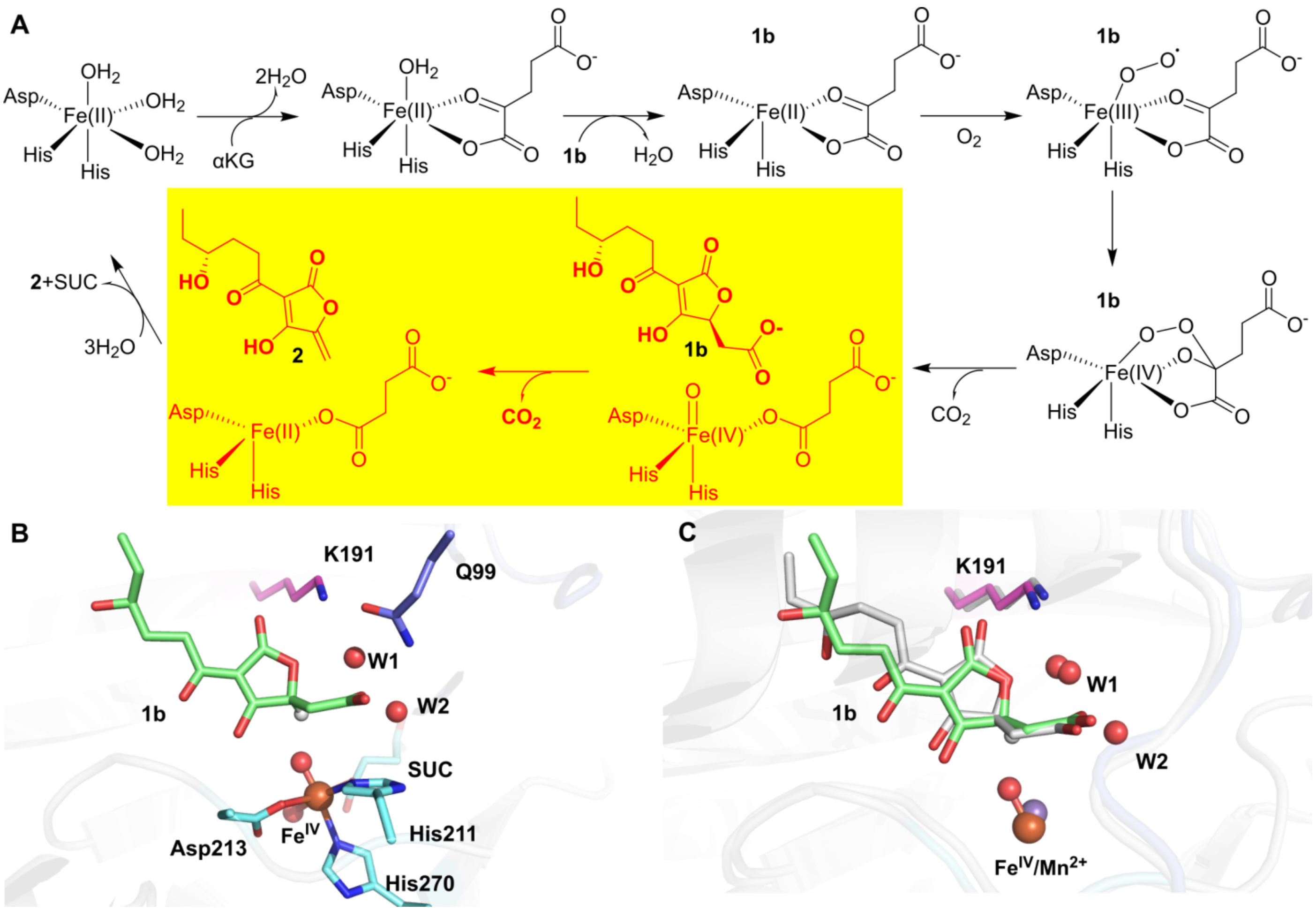

Figure S4. Proposed General Catalytic Mechanism, Key Steps Studied, and Initial Structure for QM/MM Simulations

(A) Proposed general catalytic mechanism for Fe/αKG oxygenases. The process studied in this work is highlighted in red with a yellow background. SUC represents succinate.

(B) MD-optimized structure used in QM/MM simulations of the decarboxylation process catalyzed by TraH. The QM region is highlighted in stick or ball representation. Residues coordinating iron and succinate (SUC) are shown in cyan, the lid loop residue in blue, key DSBH residue in magenta, the iron ion in orange, and water molecules in red. The substrate is displayed in green.

(C) Comparison of the structures from MD optimization and crystals, highlighting the similar placement of the water molecule W1 near K191 in both.

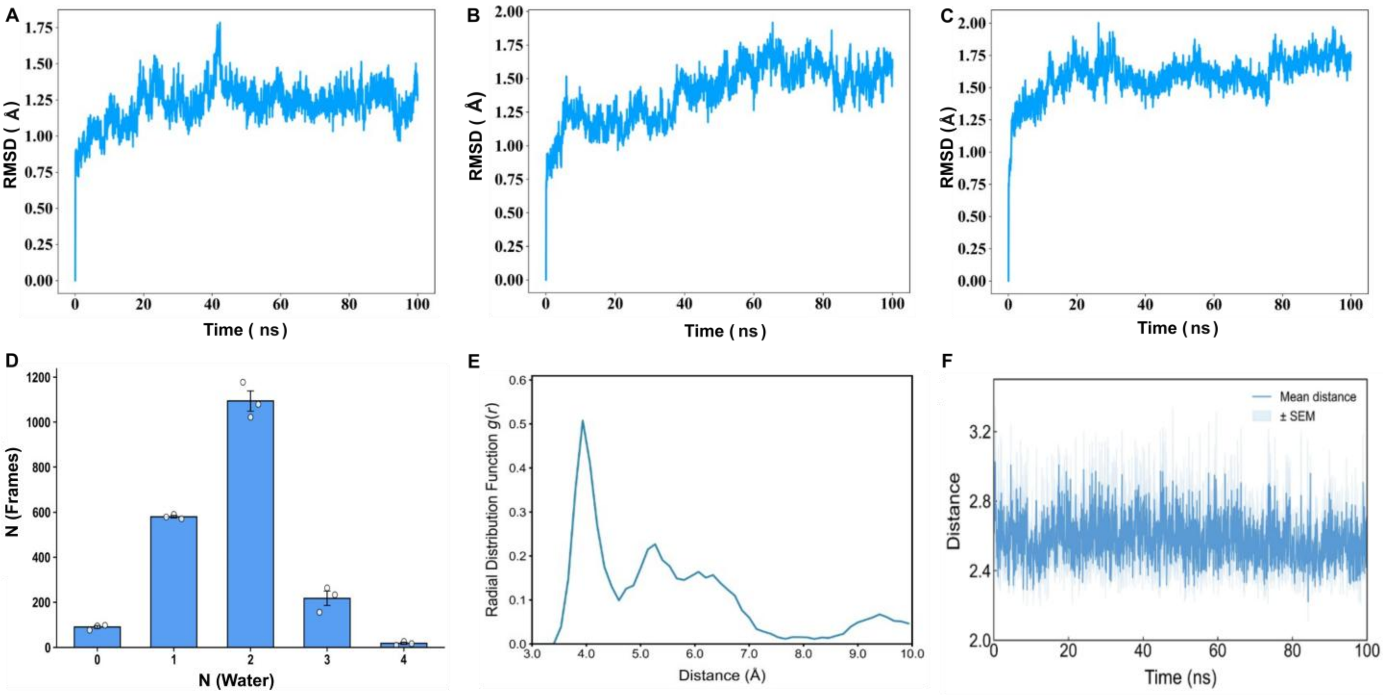

Figure S5. Molecular Dynamics Simulations Reveal Structural Dynamics and Preorganization in the TraH Catalytic Cycle

(A-C) **Backbone stability across key catalytic states.** Root mean square deviation (RMSD) of the protein backbone during MD simulations for **(A)** the reactant complex (Fe(IV)=O state), **(B)** intermediate INT1, and **(C)** intermediate INT2. Stable plateau regions in all states indicate well-equilibrated systems, providing a reliable structural foundation for subsequent mechanistic analysis. Data are from three independent trajectories.

(**D**) **Solvent organization near the catalytic base K191.** Distribution of water molecule counts within 3 Å of the K191 guanidinium group during MD simulations of the reactant complex. The prevalent presence of one or two water molecules indicates a stable, pre-organized water cluster poised to function as a proton-transfer chain. Data represent mean ± s.e.m. from three independent 100-ns trajectories.

(E) Solvation structure of the ferryl center. Radial distribution function (RDF) of water molecules around the Fe(IV)=O center in TraH was calculated over the MD trajectory. This arrangement, while not constituting direct metal coordination, delineates a pre-organized water network that could facilitate subsequent proton-transfer events following the primary HAT step.

(F) Pre-reactive geometry for hydrogen atom transfer (HAT). Distribution of the distance between the oxo oxygen (O) of Fe(IV)=O and the target hydrogen atom (H) at the C5 position of the substrate. The narrow distribution centered at 2.4-2.8 Å demonstrates that the active site pre-positions the substrate in an optimal geometry for the initial HAT step, minimizing reorganization energy. Shaded regions indicate SEM from three independent replicas.

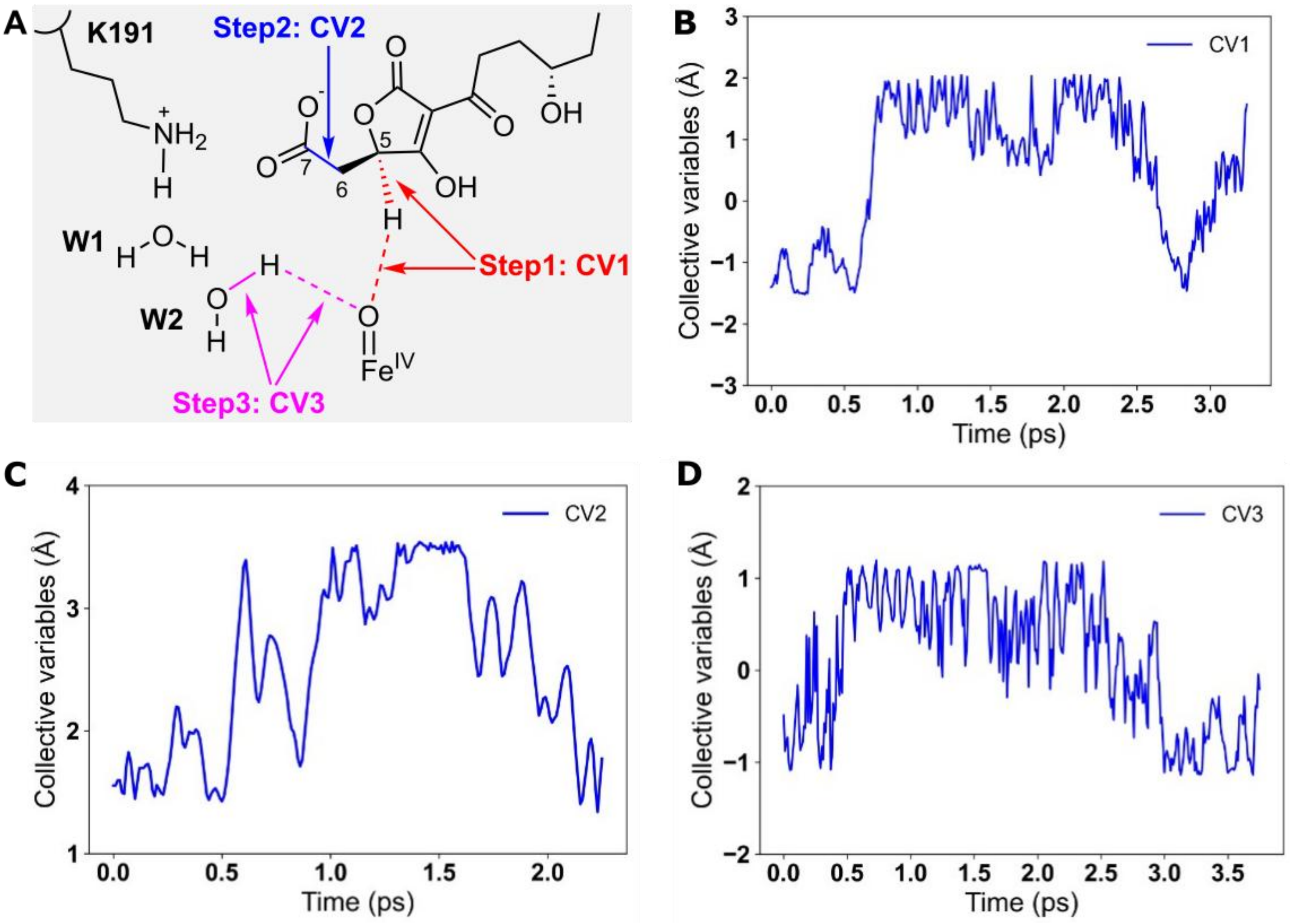

Figure S6. Collective Variables Used to Monitor the TraH-Catalyzed Decarboxylation during QM/MM Metadynamics Simulations

(A) Definition of the collective variables (CVs) used to describe the TraH-catalyzed decarboxylation of crustosic acid. CV1 represents the difference between the distance from H3 of the substrate to C3 and to O1 of the iron center. CV2 corresponds to the distance between C7 and C6 atoms of the substrate, while CV3 reflects the difference between the distance from H1 of water molecule W2 to its OW and to O1 of the iron center.

(B-D) The evolution of the collective variables during the QM/MM metadynamics simulation of the decarboxylation step catalyzed by TraH shows the dynamics of CV1, CV2, and CV3 along the reaction coordinate.

The relevant distances for the key states are provided in **Table S2**.

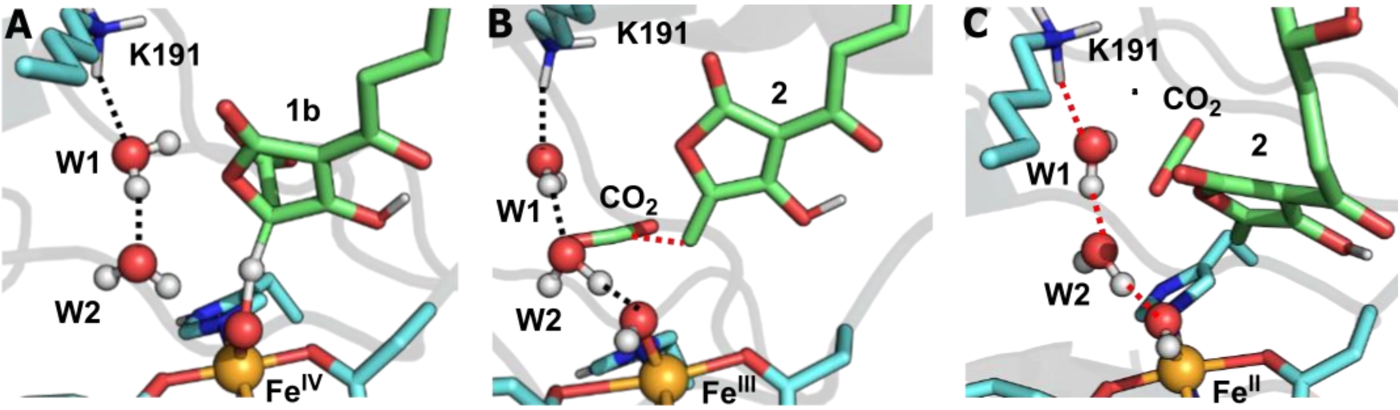

Figure S7. Structures of Transition States Along the Reaction Coordinate in TraH-Catalyzed Decarboxylation

The structures of TS1, TS2, and TS3 are shown in the left **(A)**, middle **(B)**, and right panels **(C)**, respectively. Carbon atoms of **1b** or **2** are shown in green, key residues in cyan, and the iron center in orange. Relevant hydrogen bonds are represented by dotted lines, and covalent bonds undergoing formation or cleavage are indicated by red dashed lines.

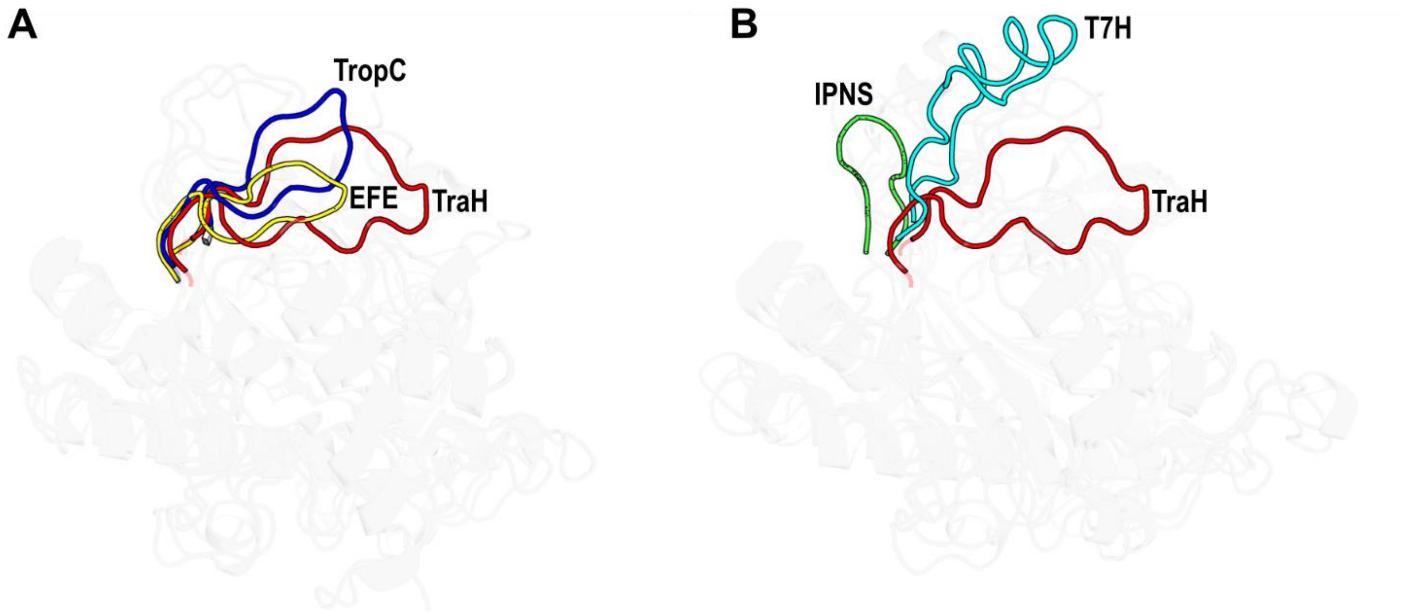

Figure S8. Structural Comparison of the Lid Loop in T7H with Its Homologs

(A) The lip loops of TraH, EFE (PDB ID: 5V2Z), TropC (PDB ID: 6XJJ), T7H (PDB ID: 5C3R), and **(B)** IPNS (PDB ID: 1BK0) are highlighted in red, yellow, blue, cyan, and green, respectively.

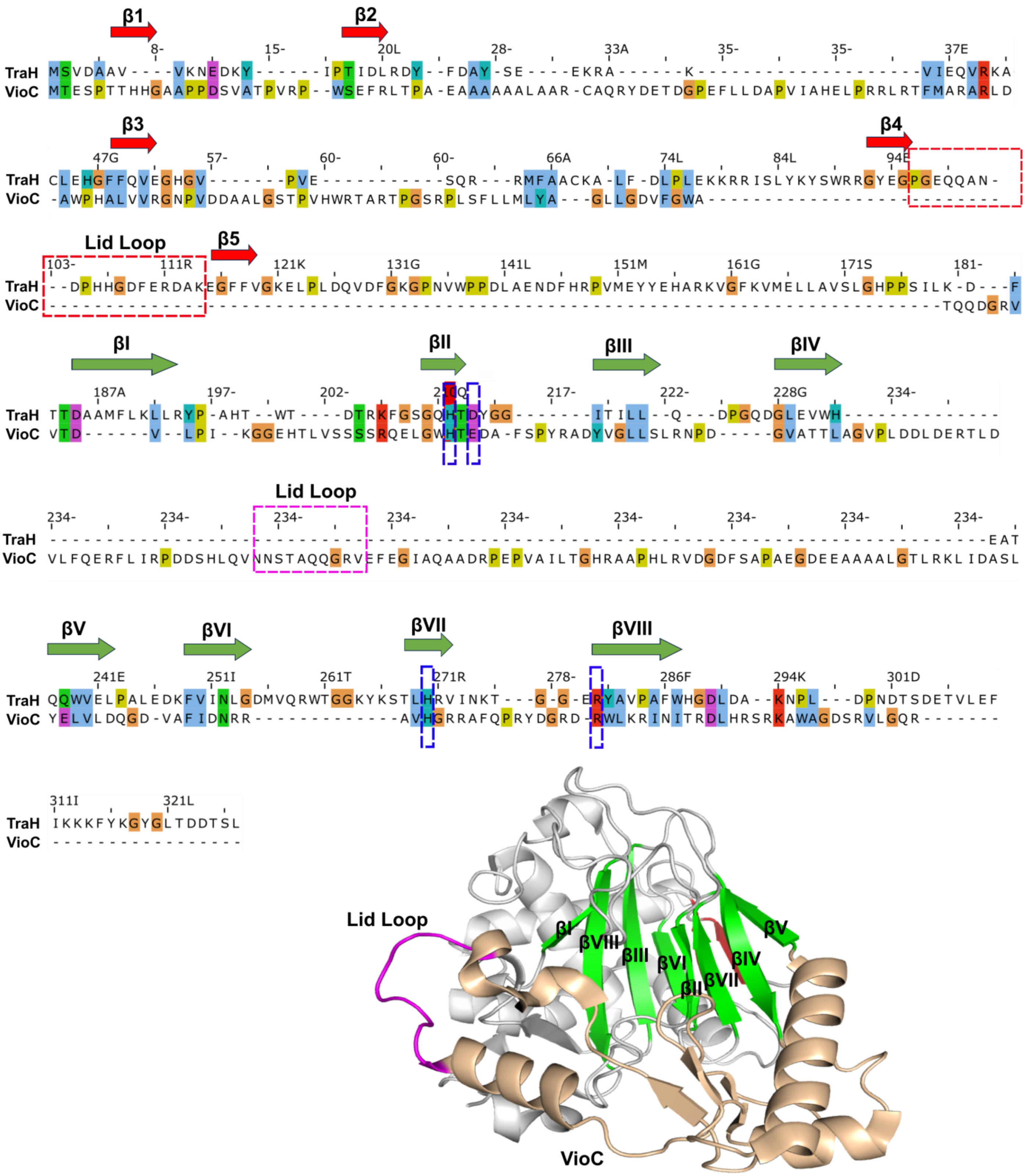

Figure S9. Sequence alignment and structural comparison of TraH and VioC

In the alignment, red and magenta boxes highlight the lid loops of TraH and VioC, respectively; blue boxes indicate conserved iron/αKG-coordinating residues. The lower right panel shows the corresponding VioC structure (PDB: 6AML), with the DBSH core in green and the central insertion (including the magenta lid loop) in wheat. This structural view of VioC is provided for comparison with the TraH structure presented in the main text (**Figure 2B**).

Cartesian Coordinates of Key Reaction State Structures from QM/MM-Based Metadynamics Simulations

RC:

Fe	10.599299	9.088426	14.326259
O	10.467209	8.334477	5.543497
O	11.863110	10.673449	14.085558
O	11.237321	11.685162	16.019356
O	9.879376	9.763557	12.998720
O	8.926932	9.759188	16.055733
O	5.355986	4.381248	12.642554
O	4.756777	4.530097	14.738863
O	9.597816	7.382253	14.235480
O	8.474546	6.788457	16.048529
O	9.312743	10.996625	8.922003
O	9.272379	14.934942	11.134830
O	10.155406	12.609668	11.962226
O	8.378717	12.558002	7.609647
O	10.940591	8.637197	8.908464
O	13.080218	9.476680	8.889229
O	9.385123	6.409021	10.435669
O	8.447258	8.119018	8.288589
N	11.807043	6.776435	6.666495
N	7.975727	8.766510	5.533771
N	13.228043	6.380080	12.227274
N	11.955712	7.973242	13.008901
N	13.417758	7.776731	17.015356
N	11.547137	8.178349	15.950683
C	11.447982	5.765635	3.640789
C	12.001376	7.093053	4.198204
C	11.344867	7.455512	5.583640
C	6.156057	10.050578	6.852426
C	6.505850	9.204697	5.571862
C	15.318155	7.553378	11.703398
C	13.938498	7.543355	12.193771
C	12.043829	6.663888	12.805615
C	13.115548	8.519559	12.606281
C	12.502725	12.907560	14.419340
C	11.788967	11.686276	14.898776
C	13.241162	5.476303	17.766249
C	12.733131	6.586344	17.015728
C	12.775232	8.633821	16.143887
C	11.660842	6.805221	16.178963
C	8.546365	6.869178	14.808545
C	7.328341	6.381974	14.014218
C	7.032212	4.889472	14.212067
C	5.608542	4.530782	13.850930
C	9.942628	10.901641	10.269741

C	8.871440	12.250935	8.677311
C	9.134879	13.052120	9.830214
C	9.737482	12.224938	10.771779
C	8.846796	14.486135	10.039465
C	8.176570	15.203292	8.965620
C	11.462418	10.556541	10.209631
C	11.878361	9.434618	9.275179
H	10.367400	5.780682	3.495810
H	11.659633	4.964608	4.377547
H	13.073620	7.117077	4.223054
H	11.714523	7.973034	3.682221
H	12.138998	5.814360	6.565512
H	11.468830	7.047712	7.583758
H	6.685517	11.008537	6.886690
H	6.269398	9.478201	7.758927
H	5.907398	8.287527	5.517668
H	6.298374	9.828640	4.700157
H	8.647305	9.533401	5.559995
H	8.178217	8.237491	6.423269
H	8.074740	8.191858	4.667647
H	15.548473	8.552597	11.372857
H	15.456043	6.781469	10.915999
H	13.622731	5.440385	12.132570
H	11.308151	5.921649	13.060480
H	13.367031	9.514853	12.869159
H	12.811134	13.484406	15.277431
H	11.761860	13.465666	13.864383
H	12.447247	4.829233	18.066925
H	13.764892	5.784403	18.669661
H	14.402265	7.974797	17.160265
H	13.178671	9.614165	15.970785
H	10.801339	6.171357	16.035660
H	9.538790	10.270586	16.604591
H	8.862689	8.899516	16.405325
H	7.543448	6.646988	12.991858
H	6.589738	7.054304	14.402611
H	7.435376	4.543033	15.137744
H	7.640842	4.386837	13.493563
H	9.368261	10.216464	10.884348
H	7.105958	15.006742	9.078644
H	8.454905	14.645168	8.064529
H	9.937837	13.578674	11.916275
H	11.712029	10.257088	11.227713
H	12.082591	11.433149	10.012346
H	9.216125	7.029500	11.147077
H	10.274158	6.605528	10.152310
H	8.538669	7.355360	8.889442

H	9.351787	8.546896	8.481151
H	12.034513	5.427496	2.691186
H	5.073669	10.322138	6.674908
H	13.398080	12.645144	13.759007
H	16.056910	7.268210	12.461717
H	13.963402	4.875570	17.179359
H	8.443022	16.228987	8.888634

TS1:

Fe	10.527956	8.980647	13.939635
O	10.417538	8.032160	5.409847
O	11.851810	10.320900	13.920869
O	12.018511	11.215848	16.002300
O	9.568985	10.063377	13.058967
O	9.413854	10.030105	15.865614
O	5.047237	4.810483	12.624246
O	5.019961	4.642262	14.841903
O	9.289276	7.525255	13.883175
O	8.103070	7.535282	15.782295
O	9.137645	10.916860	9.520049
O	10.201861	15.275064	10.248115
O	11.362799	13.188051	11.179887
O	7.795671	12.129188	8.266803
O	11.090071	9.874114	8.009495
O	13.223481	9.935363	8.894508
O	7.939590	8.624007	11.044972
O	8.958028	8.214972	8.320192
N	12.432203	7.964538	6.447178
N	7.942941	8.864359	5.412432
N	13.200176	5.937962	12.385347
N	11.914161	7.694227	12.755758
N	13.178965	7.601418	16.874765
N	11.429132	8.153416	15.685041
C	11.702118	6.153611	3.492367
C	12.236645	7.565588	3.970052
C	11.632141	7.874355	5.354368
C	6.049480	10.083713	6.503439
C	6.533924	9.272838	5.295043
C	15.405728	6.931870	11.710367
C	13.955485	7.025028	12.097359
C	11.987648	6.364709	12.771873
C	13.165407	8.110062	12.336093
C	12.043384	12.664392	14.077950
C	11.915578	11.344630	14.808024
C	12.778877	5.269195	17.676888
C	12.388521	6.442381	16.877634
C	12.641047	8.492757	16.012571

C	11.309344	6.811027	16.073366
C	8.193791	7.285327	14.558588
C	7.030816	6.582773	13.958173
C	7.133470	5.058282	13.813125
C	5.615633	4.722873	13.752307
C	10.085617	11.138642	10.575739
C	8.730323	12.069505	9.039570
C	9.614141	13.123482	9.589508
C	10.383106	12.566914	10.562737
C	9.528322	14.539294	9.479556
C	8.631996	15.243694	8.438723
C	11.418551	10.316241	10.378147
C	11.942380	10.026015	8.971601
H	10.641529	6.202368	3.253206
H	11.888804	5.473976	4.317159
H	13.312574	7.676868	4.095463
H	11.839047	8.411674	3.393996
H	13.364084	7.574926	6.528035
H	12.021414	8.364550	7.290818
H	6.644046	10.985701	6.726575
H	6.103723	9.450808	7.407612
H	6.063180	8.315628	5.290962
H	6.391602	9.778255	4.342771
H	8.589611	9.651439	5.409270
H	8.173108	8.320639	6.310680
H	8.247673	8.319497	4.545125
H	15.581901	7.886701	11.251926
H	15.547931	6.080142	11.027493
H	13.533486	4.965446	12.413857
H	11.216147	5.782549	13.205813
H	13.400722	9.152416	12.330731
H	11.878169	13.502136	14.753932
H	11.365021	12.629897	13.243073
H	11.938837	4.595949	17.886703
H	13.166048	5.551777	18.664907
H	14.164372	7.754657	17.085335
H	13.206249	9.348991	15.760342
H	10.368715	6.330985	15.882195
H	8.728165	10.689421	15.732426
H	8.992012	9.137900	15.951008
H	6.810676	6.924796	12.945320
H	6.170336	6.812459	14.559429
H	7.616360	4.552985	14.644315
H	7.705929	4.812203	12.904496
H	9.772521	10.509216	11.707151
H	7.540123	15.253855	8.716689
H	8.792792	14.704032	7.477910

H	11.174953	14.119380	10.860785
H	11.366378	9.388911	10.989884
H	12.225078	10.874283	10.862161
H	8.594340	8.865041	11.696320
H	7.827261	7.687756	11.200175
H	8.654125	8.461396	9.207314
H	9.731256	8.819264	8.176224
H	12.319818	5.746905	2.653762
H	4.958189	10.332628	6.384866
H	13.084002	12.784988	13.640265
H	16.167158	6.910046	12.569859
H	13.627638	4.625799	17.294468
H	8.975768	16.240068	8.362484

INT1:

Fe	10.547531	8.920685	14.244973
O	10.561508	8.507208	5.571428
O	11.695068	10.385633	13.939270
O	12.044082	11.152018	16.043722
O	9.559114	9.798003	12.890077
O	9.687239	10.039955	16.081692
O	4.701915	5.072738	12.875170
O	4.822119	4.354641	15.011064
O	9.194882	7.568061	14.153754
O	7.976270	7.967669	16.000504
O	9.075965	11.235657	9.322117
O	9.355455	15.497819	10.560853
O	11.034927	13.634233	11.143220
O	7.555934	12.395402	8.078177
O	11.361516	8.914038	8.848547
O	13.049175	10.483225	9.090445
O	7.976213	8.710110	11.164050
O	8.633646	8.499545	8.244879
N	11.589678	6.822874	6.697791
N	7.899256	9.469100	5.584811
N	13.367653	6.020511	12.848965
N	12.002697	7.694112	13.129232
N	13.091904	7.559454	17.133768
N	11.440244	8.125074	15.891731
C	11.356265	5.933233	3.647576
C	12.134458	7.083083	4.306286
C	11.393948	7.579853	5.603491
C	5.879628	10.066790	6.763706
C	6.373959	9.292014	5.500923
C	15.360592	7.098250	11.827686
C	13.981003	7.089264	12.319569
C	12.187608	6.426290	13.337911

C	13.154113	8.137955	12.488280
C	12.278477	12.705636	14.253683
C	11.923487	11.354379	14.838543
C	12.512002	4.974620	17.569979
C	12.256897	6.408533	17.086033
C	12.619619	8.513509	16.284227
C	11.230909	6.788094	16.212887
C	8.052461	7.576955	14.858477
C	6.874730	7.073450	14.079284
C	6.632987	5.549848	14.141689
C	5.210680	5.012860	13.995826
C	10.177436	11.495742	10.118929
C	8.487680	12.431762	8.863787
C	9.161800	13.492317	9.577239
C	10.229462	12.905149	10.326282
C	8.938589	14.875617	9.559787
C	8.257268	15.513357	8.396306
C	11.020723	10.447926	10.555184
C	11.962151	9.908805	9.288441
H	10.348333	6.310895	3.480793
H	11.347235	5.002454	4.205041
H	13.124696	6.775454	4.529799
H	12.088973	7.927797	3.637519
H	12.158636	5.993525	6.613265
H	11.444308	7.301206	7.575427
H	6.213379	11.096937	6.829571
H	6.142346	9.444378	7.627779
H	6.181400	8.202124	5.496181
H	6.057360	9.737030	4.549750
H	8.230924	10.448174	5.589421
H	8.172843	9.080531	6.532182
H	8.446563	9.028353	4.812380
H	15.400797	7.982035	11.212566
H	15.508395	6.268957	11.143723
H	13.726606	5.076687	12.860007
H	11.523092	5.764320	13.843898
H	13.296226	9.123725	12.105065
H	12.247290	13.443252	15.046762
H	11.504117	13.045863	13.566415
H	11.572396	4.458879	17.317812
H	12.651844	5.004163	18.655445
H	14.087105	7.557412	17.338078
H	13.158071	9.409382	16.066654
H	10.239348	6.427431	16.039566
H	9.521383	10.995014	16.123127
H	8.845099	9.568944	16.191973
H	6.775193	7.546689	13.100505

H	6.103870	7.575632	14.680043
H	6.987524	5.161398	15.106754
H	7.180560	5.056559	13.320256
H	9.383568	10.637446	13.196032
H	7.230032	15.718018	8.686852
H	8.225124	14.752460	7.630951
H	10.546259	14.499128	11.162781
H	10.528937	9.537568	10.855648
H	11.671897	10.757418	11.385053
H	8.608717	9.121378	11.834699
H	8.418042	7.879049	10.960911
H	8.473975	8.837054	9.168437
H	9.585974	8.539570	8.200650
H	11.901029	5.728695	2.703356
H	4.813009	10.114670	6.670033
H	13.230008	12.707685	13.646420
H	16.253138	7.084536	12.578915
H	13.477924	4.463450	17.262321
H	8.718215	16.395557	8.039948

TS2:

Fe	10.350999	8.643500	14.286999
O	10.809999	8.036500	5.628000
O	11.400000	10.268500	14.155999
O	11.773999	11.437500	16.034000
O	8.932000	9.082500	13.055000
O	9.125000	9.613500	16.084000
O	4.818000	4.629500	12.712000
O	4.631000	4.241500	14.950999
O	9.327001	6.758500	14.167999
O	8.146000	7.152500	16.064999
O	8.645000	11.084499	9.280000
O	10.334001	15.208500	9.747999
O	11.214000	13.102500	10.788999
O	7.275000	12.549500	8.112000
O	11.487999	7.765500	9.618000
O	13.072000	9.425499	9.554000
O	8.752000	7.313499	10.907000
O	8.934999	7.102499	7.981999
N	11.909000	6.359499	6.743000
N	7.934000	8.348499	5.650000
N	13.026999	6.095500	12.381000
N	11.687000	7.759500	12.942999
N	13.344999	7.454499	16.967999
N	11.433001	7.745500	15.915999
C	11.914999	5.129499	3.724000
C	11.967999	6.614500	4.146999

C	11.529000	7.035500	5.634000
C	6.097000	9.773499	6.750999
C	6.467000	8.820499	5.573000
C	15.249999	7.251500	11.835999
C	13.763000	7.266500	12.165999
C	11.801999	6.453500	12.790000
C	12.922999	8.277500	12.494999
C	12.212999	12.547501	13.916999
C	11.724000	11.386499	14.793999
C	13.170000	4.902500	17.591000
C	12.708000	6.180500	16.921999
C	12.549999	8.337500	16.226999
C	11.565999	6.363500	16.214998
C	8.183000	6.857500	14.843999
C	6.817000	6.528500	14.203000
C	6.729000	4.990500	14.098000
C	5.242000	4.630500	13.906000
C	9.787000	11.244499	10.106000
C	8.191000	12.407499	8.929999
C	9.143999	13.313499	9.493999
C	10.073000	12.570499	10.182999
C	9.338999	14.718499	9.246000
C	8.361000	15.374499	8.323999
C	10.584999	10.160500	10.425000
C	12.158999	8.718499	9.610999
H	10.867000	4.817500	3.660000
H	12.347999	4.479500	4.478000
H	12.894999	7.117500	3.826000
H	11.127000	7.101500	3.659000
H	12.136000	5.369500	6.709000
H	11.672999	6.669499	7.680000
H	6.621000	10.713499	6.796000
H	6.254000	9.298499	7.724000
H	5.895999	7.899500	5.580000
H	6.311999	9.361500	4.622000
H	8.669000	9.088500	5.640000
H	8.063000	7.885499	6.578000
H	8.125000	7.680500	4.882000
H	15.478000	8.178499	11.322000
H	15.459999	6.402500	11.164000
H	13.332999	5.115500	12.268999
H	11.023999	5.819499	13.134999
H	13.131000	9.330500	12.570999
H	12.346000	13.385500	14.618999
H	11.445999	12.796500	13.163000
H	12.339000	4.197500	17.448000
H	13.441999	4.920500	18.652000

H	14.261000	7.536500	17.369999
H	12.783999	9.361500	16.052999
H	10.756000	5.669500	16.132999
H	8.253000	10.015499	16.057999
H	8.921000	8.693500	16.282999
H	6.740000	7.064499	13.278999
H	6.055000	6.907499	14.884000
H	7.071000	4.445500	14.931999
H	7.288000	4.654500	13.210999
H	8.594999	9.953500	12.891999
H	7.318000	15.180500	8.618000
H	8.502999	14.862500	7.348000
H	11.236000	14.048499	10.421000
H	10.151999	9.220499	10.566000
H	11.407000	10.308499	11.164000
H	8.991999	7.838500	11.738999
H	8.790000	6.387500	11.191999
H	8.815000	7.245500	8.959000
H	9.659999	7.703499	7.689000
H	12.414275	4.936021	2.726898
H	5.033232	9.979297	6.608971
H	13.176767	12.488804	13.419174
H	16.062319	7.174688	12.692521
H	13.996812	4.377862	17.170710
H	8.542159	16.423050	8.208782

INT2:

Fe	10.528766	8.653749	14.853255
O	11.314479	7.579588	6.949342
O	12.054278	10.429723	14.681525
O	11.281404	11.846594	16.316721
O	9.654709	9.466710	13.249988
O	9.706590	9.860465	16.403698
O	4.570612	5.668696	12.738226
O	4.587470	5.572460	14.967144
O	9.258351	6.982667	14.827914
O	8.052162	7.975992	16.414511
O	13.508878	7.685294	8.733191
O	11.840569	8.133802	10.271894
O	9.588102	11.989227	10.395761
O	10.463634	16.173012	11.513109
O	11.653629	13.971197	12.485801
O	8.261866	13.329868	9.211632
O	8.221360	8.373005	11.104509
O	8.953482	8.696637	8.540416
N	11.949541	5.733334	5.880095
N	8.102912	9.460683	5.883686

N	12.931192	5.561391	12.743344
N	11.840987	7.271760	13.595897
N	13.534011	6.886907	17.332542
N	11.648596	7.349618	16.298250
C	10.985876	6.664902	3.134395
C	11.504033	7.624426	4.384434
C	11.505396	7.026183	5.877398
C	6.364885	10.673059	7.216496
C	6.631685	9.826329	5.918307
C	15.133399	6.590350	12.039448
C	13.786517	6.675086	12.668641
C	11.810489	5.975650	13.401231
C	13.087562	7.699581	13.202753
C	13.209792	12.522153	15.185887
C	12.084555	11.459418	15.399493
C	13.244723	4.503648	17.908237
C	12.762529	5.720330	17.241405
C	12.861766	7.785105	16.543364
C	11.618611	5.996869	16.512049
C	8.179232	7.467987	15.282026
C	6.962370	7.242318	14.397631
C	6.683082	5.799562	13.880369
C	5.126361	5.652906	13.844702
C	12.667915	7.917268	9.495114
C	10.615567	12.054478	11.363541
C	9.059350	13.230642	10.102637
C	9.770184	14.166933	10.872605
C	10.763024	13.435209	11.654582
C	9.707037	15.550094	10.756115
C	8.877545	16.239065	9.683418
C	11.237962	10.907636	11.813558
H	10.236957	7.204667	2.561467
H	10.477990	5.808831	3.538428
H	12.557910	7.893876	4.322649
H	10.923049	8.549122	4.388049
H	12.230186	5.301703	4.986097
H	12.026778	5.202713	6.762646
H	7.048550	11.537072	7.108727
H	6.642852	10.146269	8.117493
H	6.170763	8.856215	5.926583
H	6.475246	10.331433	4.949578
H	8.699303	10.315377	5.905972
H	8.398932	8.957685	6.721753
H	8.229262	9.017599	4.968125
H	15.192380	7.485089	11.430369
H	15.044452	5.791898	11.309111
H	13.241626	4.589703	12.665984

H	10.967589	5.366622	13.645079
H	13.376635	8.730502	13.297256
H	13.637297	12.732455	16.178875
H	12.667025	13.418365	14.863210
H	12.447591	3.756292	18.039282
H	13.598007	4.750375	18.917097
H	14.512765	6.957530	17.611801
H	13.204239	8.808556	16.360355
H	10.729476	5.475227	16.247665
H	9.177785	10.297528	13.320455
H	9.868094	10.821609	16.465082
H	8.781683	9.488785	16.506763
H	6.975592	8.084641	13.685712
H	6.140965	7.434162	15.058180
H	7.025113	5.079836	14.626132
H	7.138209	5.609488	12.929557
H	8.946861	17.342554	9.870309
H	7.817540	16.014662	9.736144
H	11.599939	14.962794	12.366009
H	11.044628	9.989322	11.302118
H	11.772820	10.863852	12.729918
H	8.868567	8.749695	11.690342
H	7.808218	7.912294	11.833883
H	8.546919	8.611527	9.415940
H	9.864739	8.455488	8.749479
H	11.672095	6.140891	2.372444
H	5.357294	11.017407	7.274331
H	14.017109	12.267037	14.489026
H	16.072521	6.373878	12.674104
H	13.981576	3.890458	17.388243
H	9.212117	16.153223	8.628317

TS2:

Fe	10.640717	8.681531	14.744364
O	10.778953	7.423833	6.900864
O	12.212204	10.311474	14.911999
O	11.221109	11.796738	16.390877
O	9.537176	9.712793	13.158080
O	9.730535	9.742456	16.419024
O	4.780278	5.628533	12.705629
O	4.683711	5.634115	14.979463
O	9.247040	7.208333	14.864081
O	7.870038	8.084108	16.367830
O	13.842113	7.864318	8.771432
O	11.938562	7.848569	10.139134
O	9.640936	12.009046	10.114198
O	10.617583	16.249470	11.579339

O	11.730991	14.059855	12.096916
O	7.946278	13.293247	9.480550
O	9.131453	8.385618	11.175175
O	9.223636	8.956788	8.561255
N	11.931556	5.732539	5.925434
N	8.447820	9.449429	5.892706
N	13.124163	5.712562	13.184679
N	11.781913	7.415197	13.492527
N	13.695939	6.896712	17.366001
N	11.899631	7.498633	16.228846
C	10.953785	6.576433	3.128959
C	11.395205	7.514269	4.405537
C	11.291500	6.919981	5.891193
C	6.648435	10.794487	7.208662
C	6.991466	9.981954	5.890127
C	15.238666	6.660089	12.177016
C	13.859354	6.829601	12.783764
C	11.897886	6.087332	13.574848
C	12.983939	7.857655	12.974174
C	13.012202	12.519135	15.053943
C	12.095427	11.424014	15.509620
C	13.304544	4.471606	17.909706
C	12.882084	5.742635	17.225046
C	13.092514	7.849080	16.579233
C	11.804204	6.143663	16.474136
C	8.064922	7.576816	15.239911
C	6.881927	7.437778	14.331628
C	6.749885	5.889615	13.997571
C	5.289122	5.661977	13.861712
C	12.880857	7.844668	9.440551
C	10.740932	12.135067	10.996706
C	9.066382	13.250472	9.916715
C	9.992520	14.261570	10.464684
C	10.912094	13.489743	11.201200
C	9.918674	15.688619	10.710589
C	8.924154	16.348566	9.813277
C	11.366055	11.066445	11.557220
H	10.120259	7.064080	2.638278
H	10.485557	5.674376	3.569922
H	12.473701	7.739988	4.407773
H	10.850218	8.455700	4.370616
H	12.086429	5.225874	5.051978
H	11.942432	5.223348	6.822098
H	7.218493	11.717235	7.345504
H	6.793953	10.143272	8.082358
H	6.342945	9.130604	5.878691
H	6.805708	10.476878	4.921025

H	9.096899	10.262457	5.848174
H	8.643515	9.014388	6.819504
H	8.585562	8.863404	5.021183
H	15.534316	7.584907	11.648904
H	15.270483	5.802881	11.506739
H	13.544709	4.807026	13.441097
H	11.113074	5.499399	13.989406
H	13.222724	8.901858	12.934489
H	13.201656	13.134702	15.932863
H	12.438626	13.178711	14.409320
H	12.471573	3.837270	18.167423
H	13.752911	4.770600	18.866816
H	14.697061	6.867869	17.533701
H	13.532685	8.823949	16.370989
H	10.871595	5.655446	16.325621
H	8.988360	10.342083	13.653836
H	9.957655	10.731781	16.393639
H	8.819784	9.368854	16.469513
H	7.071201	7.963230	13.413142
H	5.987370	7.809640	14.815796
H	7.249675	5.314664	14.772806
H	7.240806	5.752211	13.055169
H	8.993154	17.407652	10.016269
H	7.888961	16.100460	10.022612
H	11.390999	14.966841	12.254042
H	10.982435	10.059490	11.361557
H	12.234895	11.126374	12.184018
H	9.110673	9.000070	12.073936
H	9.295257	7.488194	11.450961
H	9.012653	8.793383	9.542130
H	10.052681	8.445158	8.586512
H	11.703013	6.213993	2.407383
H	5.554182	11.134482	7.131038
H	13.934705	12.275893	14.537907
H	16.019552	6.406882	12.860735
H	14.070107	3.834462	17.464203
H	9.173631	16.187229	8.738339

P:

Fe	10.696003	8.331671	14.829789
O	11.665523	7.714383	6.676073
O	12.352029	10.041190	14.655120
O	11.262244	11.420417	16.087461
O	9.251039	9.765431	13.191413
O	9.673380	9.496424	16.073503
O	4.777512	5.738312	12.189424
O	4.875767	5.524092	14.499576

O	9.319018	6.936642	14.928499
O	7.542431	7.878780	16.029850
O	13.102840	7.417867	9.670508
O	10.742697	7.315444	9.604617
O	9.597062	11.896065	9.733584
O	10.726489	16.203041	10.412894
O	11.736575	14.125014	11.485055
O	8.116441	13.112607	8.596772
O	7.719208	9.486505	10.685462
O	8.164284	8.435042	8.153381
N	11.298566	5.741524	5.546255
N	7.720366	9.666478	5.595919
N	13.362276	5.739020	12.981342
N	11.918924	7.412272	13.073584
N	13.756590	6.879427	17.175768
N	11.998844	7.300337	15.943182
C	11.579506	7.308093	2.764485
C	11.828088	7.759704	4.301236
C	11.621503	7.058817	5.609925
C	5.770876	10.778340	6.923503
C	6.267729	10.056182	5.619019
C	15.488325	6.804695	12.351968
C	14.128686	6.887621	12.942885
C	12.067863	6.076815	13.008083
C	13.206207	7.888589	13.065894
C	13.013023	12.391760	14.840473
C	12.138812	11.151566	15.223516
C	13.250576	4.494720	17.957315
C	12.950484	5.711469	17.172672
C	13.137705	7.778852	16.310537
C	11.939219	5.952655	16.258539
C	8.107015	7.336328	15.013844
C	7.342152	7.191953	13.690843
C	6.938317	5.728458	13.216338
C	5.337212	5.639380	13.294652
C	11.920059	7.298599	9.635324
C	10.478947	12.140034	10.807302
C	9.118933	13.101129	9.301421
C	9.904834	14.161614	9.898069
C	10.775231	13.538279	10.765578
C	9.856022	15.579224	9.723280
C	8.901709	16.271780	8.760879
C	11.159047	11.227547	11.503824
H	11.818337	8.288708	2.354658
H	10.471207	7.251987	2.681118
H	12.838267	8.167856	4.405834
H	11.252671	8.690906	4.310450

H	11.343429	5.187052	4.673568
H	11.295202	5.227167	6.413610
H	6.398240	11.592575	7.286599
H	5.866707	10.099494	7.762661
H	5.732045	9.141082	5.541137
H	6.140755	10.638859	4.732211
H	8.261016	10.524631	5.584532
H	8.010319	8.988023	7.306330
H	7.833569	9.238516	4.675368
H	15.663352	7.811068	11.983997
H	15.431984	6.077647	11.524837
H	13.756361	4.804183	12.879739
H	11.302709	5.362588	12.931835
H	13.256847	8.955026	13.098760
H	13.206569	12.956914	15.763745
H	12.367111	13.014291	14.224201
H	12.385827	3.856236	18.122526
H	13.636071	4.803998	18.917070
H	14.774512	6.921095	17.328602
H	13.479237	8.754697	15.995515
H	11.009716	5.483467	16.025820
H	8.677093	9.947309	13.940742
H	10.089311	10.421850	16.084881
H	8.717649	9.254336	16.074533
H	7.976021	7.634997	12.922823
H	6.438065	7.797029	13.764399
H	7.284884	4.991678	13.913569
H	7.296715	5.593162	12.171894
H	9.147093	17.367397	8.897659
H	7.837708	16.055933	8.961603
H	11.703701	15.040803	11.206909
H	11.091328	10.166598	11.421202
H	11.867689	11.556296	12.255226
H	8.602705	9.648977	12.470120
H	7.197721	8.681697	10.741145
H	7.800218	9.507092	9.633504
H	9.110625	8.498866	8.294663
H	12.183982	6.515996	2.270602
H	4.703231	11.133219	6.920254
H	14.027527	12.232394	14.398625
H	16.368143	6.527223	12.985882
H	14.018764	3.869996	17.511700
H	9.063771	16.122406	7.669086
