# Supplementary material for: Lid loop-mediated proton transfer revealed in the Fe/αKG-dependent decarboxylase TraH
Source: Commun Chem. 2026 Apr 14;9:159. doi: 10.1038/s42004-026-01986-9 (PMC13083874; doi:10.1038/s42004-026-01986-9)
Supplement: Supplementary file 3 — Description of Additional Supplementary Files [file 42004_2026_1986_MOESM3_ESM.pdf]

Description of Additional Supplementary Files:

File: Supplementary Data 1

Description: PDB file for 9IG5 (TraH- Mn-*α*KG-**1b**)

File: Supplementary Data 2

Description: PDB file for 9IG4 (TraH- Mn)

File: Supplementary Data 3

Description: PDB file for 9IG3 (TraH- Mn-*α*KG-**3b**)

File: Supplementary Data 4

Description: Source data file for the Figures

File: Supplementary Data 5

Description: Detailed data file for QM/MM analysis.

File: Supplementary Data 6

Description: Initial and final configurations of MD trajectories.

File: Supplementary Data 7

Description: Datafiles for phylogenetic analysis
